# Diversity between mammalian tolloid proteinases: Oligomerisation and non-catalytic domains influence activity and specificity

**DOI:** 10.1038/srep21456

**Published:** 2016-02-23

**Authors:** Christopher P. Bayley, Hilda D. Ruiz Nivia, Rana Dajani, Thomas A. Jowitt, Richard F. Collins, Heather Rada, Louise E. Bird, Clair Baldock

**Affiliations:** 1 Wellcome Trust Centre for Cell-Matrix Research, University of Manchester, Manchester, M13 9PT, UK; 2 Faculty of Life Sciences, University of Manchester, Manchester, M13 9PT, UK; 3 OPPF-UK, Research Complex at Harwell, Rutherford Appleton Laboratory, Oxford, OX11 0FA, UK

## Abstract

The mammalian tolloid family of metalloproteinases is essential for tissue patterning and extracellular matrix assembly. The four members of the family: bone morphogenetic protein-1 (BMP-1), mammalian tolloid (mTLD), tolloid-like (TLL)-1 and TLL-2 differ in their substrate specificity and activity levels, despite sharing similar domain organization. We have previously described a model of substrate exclusion by dimerisation to explain differences in the activities of monomeric BMP-1 and dimers of mTLD and TLL-1. Here we show that TLL-2, the least active member of the tolloid family, is predominantly monomeric in solution, therefore it appears unlikely that substrate exclusion via dimerisation is a mechanism for regulating TLL-2 activity. X-ray scattering and electron microscopy structural and biophysical analyses reveal an elongated shape for the monomer and flexibility in the absence of calcium. Furthermore, we show that TLL-2 can cleave chordin *in vitro*, similar to other mammalian tolloids, but truncated forms of TLL-2 mimicking BMP-1 are unable to cleave chordin. However, both the N- and C-terminal non-catalytic domains from all mammalian tolloids bind chordin with high affinity. The mechanisms underlying substrate specificity and activity in the tolloid family are complex with variation between family members and depend on both multimerisation and substrate interaction.

BMP-1 (procollagen C-proteinase-1) and mammalian tolloid (mTLD) are alternatively spliced products of the *Bmp1* gene^1^. Together with mammalian tolloid like-1 (TLL-1) and -2, they comprise a small group of zinc and calcium dependent proteinases, fundamental to morphogenesis and extracellular matrix (ECM) assembly. The BMP-1/TLD family is conserved in species ranging from *Drosophila* to humans and their importance is highlighted by the embryonic lethal phenotype of *Bmp1/Tll1* homozygous null mice, which display heart malformations and abnormal procollagen processing^2^. In vertebrates, BMP-1/TLD proteinases are involved in the biosynthetic processing of a wide range of ECM precursors including collagens, the crosslinking enzymes lysyl oxidase (LOX) and LOX-like, laminin basement membrane proteins and small leucine-rich proteoglycans osteoglycin and probiglycan (for reviews see^3^,^4^). BMP-1/TLD proteinases also release a number of TGFβ superfamily members from their corresponding latent complexes, including BMP-2 and -4, growth and differentiation factors 8 (promyostatin) and 11 and TGFβ1. This modulates dorsal ventral patterning, growth of skeletal muscle and neural tissue, and cellular behaviour respectively^5^,^6^,^7^,^8^. These dual roles have fuelled speculation that BMP-1/TLD proteinases orchestrate ECM assembly via signalling by TGF-β-like proteins^9^.

BMP-1/TLD proteinases have an N-terminal protease domain related to the digestive enzyme astacin^10^ followed by non-catalytic CUB (Complement/Uegf/BMP-1) and EGF-like domains (Fig. 1A). mTLD, TLL-1 and TLL-2 have 5 CUB and 2 EGF-like domains whereas BMP-1 lacks two CUB and one EGF C-terminal domains. BMP-1 has the highest activity whereas its splice variant mTLD has much lower activity and TLL-2 is generally accepted to have the lowest activity of the mammalian family members. BMP-1, mTLD and TLL-1 are highly expressed in developing skeletal tissue, whereas, TLL-2 has specific expression profiles in developing skeletal muscle and in the central nervous system^11^. All members of the mammalian tolloid family are capable of cleaving pro-myostatin but TLL-2 efficiently cleaves pro-myostatin *in vitro*, despite having low activity for other tolloid substrates (i.e. procollagen and chordin)^8^ and muscle mass is increased in TLL-2 deficient mice consistent with decreased levels of processed myostatin^12^. TLL-2 has also recently been shown to have a genetic association with bipolar disorder, although the mechanism is not yet understood^13^.

There is evidence that the non-catalytic domains have a negative regulatory role^14^,^15^ and we have previously provided an explanation as to why mTLD is a less efficient proteinase relative to BMP-1^16^. Our findings illustrated that BMP-1 is a monomer whereas mTLD and TLL-1 form dimers in the presence of calcium-ions, which restricts their activities by a substrate exclusion mechanism^16^,^17^. The C-terminal non-catalytic domains mediate dimerisation and removal of these domains increases the activity of mTLD and TLL-1. However, we recently showed that *Drosophila* tolloid is a monomer^18^, despite having high activity for its substrate Sog and removal of the C-terminal domains resulted in loss of activity, suggesting that there may be differences in the requirement of the non-catalytic domains and dimerisation between tolloids.

In the present report, we provide evidence that TLL-2 is predominantly monomeric in the presence of calcium. We show that truncated forms of TLL-2, having the same domain structure as BMP-1, are unable to cleave the tolloid substrate, chordin. We also demonstrate that all mammalian tolloids bind chordin with high affinity via the N-terminal and C-terminal non-catalytic domains.

## Results

### TLL-2 is preferentially monomeric in solution unlike mTLD and TLL-1

**Figure 1:**
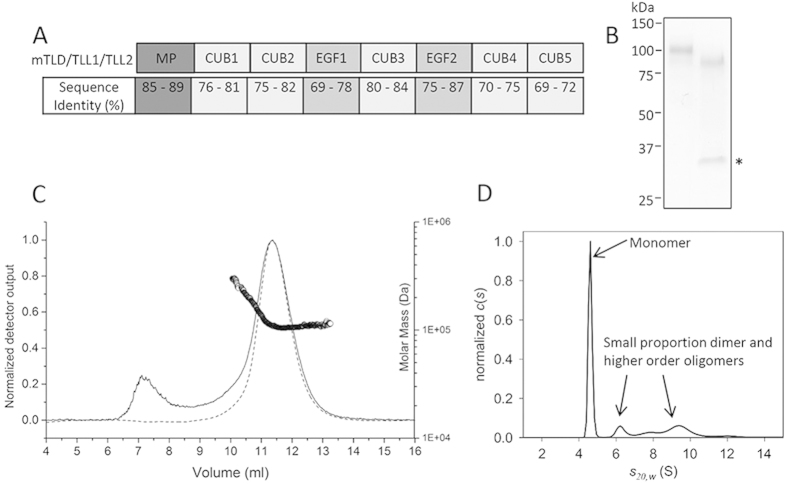
Expression and characterization of human TLL-2 which is predominantly a monomer in the presence of Ca^2+^. (**A**) Schematic diagram of the domain organisation of mTLD, TLL-1 and TLL-2 (MP = metalloproteinase domain) and sequence identities between domains in mTLD, TLL-1 and TLL-2. (**B**) Coomassie stained SDS-PAGE of purified TLL-2, in the absence and presence of PNGaseF. TLL-2 runs as a single band of >100 kDa and has a shift in mobility after treatment with PNGaseF. The PNGaseF can be seen on the gel as a single band at ~30 kDa (highlighted with an asterisk). (**C**) Multi-angle light scattering (MALS) analysis of TLL-2, the graph shows the normalised differential refractive index (dashed line), light scattering at 90 degrees (solid line) and molecular mass of the proteins as they elute from the gel filtration column. The majority of the protein elutes as a monomeric protein with an apparent molecular mass of 106.7 ± 0.97 kDa (experimental errors from polydispersity). A small amount of higher molecular weight species co-elutes at the beginning of the protein peak which could be a mixture of dimer and higher molecular weight species. (**D**) C(s) analysis of TLL-2 as derived from sedimentation velocity AUC analysis, indicating the majority of the sample is comprised of a monomer, with a small proportion of higher molecular weight species. Biophysical analyses were carried out in the presence of 2 mM calcium chloride.

Human TLL-2 was expressed in a mammalian expression system and purified as a secreted protein (Fig. 1B). Tryptic peptide analysis by MS validated the identity of the purified protein. The size and oligomeric status of TLL-2 was first analysed by multi-angle light scattering (MALS) in conjunction with size exclusion chromatography. In the presence of 2 mM calcium chloride, the majority of the protein elutes as a monomeric species with molecular mass of 106.7 ± 0.97 kDa (Fig. 1C), which is slightly larger than the predicted mass of a monomer (98.58 kDa). A small amount of higher molecular weight species co-elutes at the beginning of the protein peak which could be a mixture of dimer or higher molecular weight species. TLL-2 has 5 N-linked glycans predicted from primary sequence and digestion with PnGaseF, an amidase that cleaves between the innermost GlnAc and Asp residues of N-linked glycoproteins, yielded a shift in mobility on SDS-PAGE consistent with glycosylation (Fig. 1B). In-line quasi-elastic light scattering, which measures the diffusion of particles and therefore the hydrodynamic radius (*R*_*H*_) and sedimentation velocity analytical ultracentrifugation (AUC) which measures the sedimentation coefficient, were used to help determine the solution properties of monomeric mTLL2. A *R*_*H*_ of 4.8 ± 0.21 nm and a sedimentation coefficient of 4.78 S (Fig. 1D) are consistent with a slightly elongated monomeric protein. This is also consistent with the frictional ratio (f/f_0_) determined by AUC of 1.62 which is characteristic of a folded, but slightly extended molecule. Although the main species was a monomer both the MALS and AUC showed evidence of small amounts of higher order oligomers.

### The structure of TLL-2 is consistent with the other mammalian tolloids and stabilised by Ca^2+^

**Figure 2:**
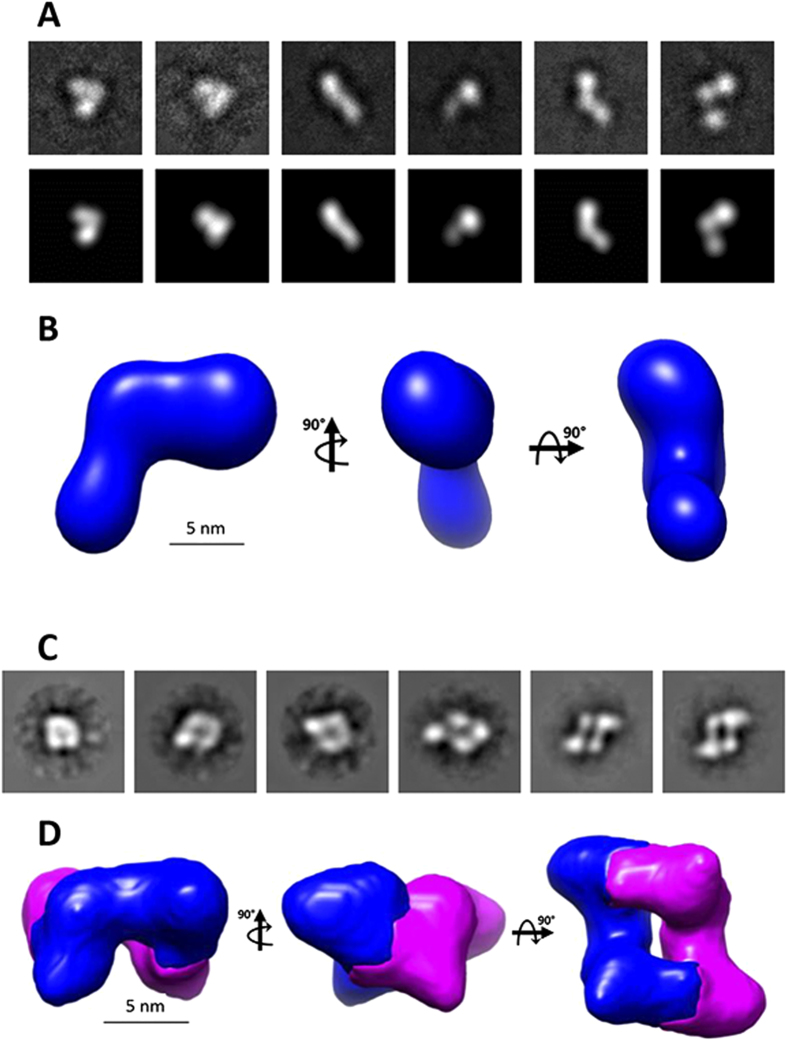
Monomeric and dimeric forms of TLL-2 can be resolved by electron microscopy. Low dose negatively stained EM images were recorded on a Tecnai T12 TEM using a CCD detector. (**A**) Selected representative class sum images (top row) and reprojections (bottom row) of different views of TLL-2 monomer (box size = 36 × 36 nm) and dimer (box size = 39 × 39 nm) (**C**). (**B**) Orthogonal views of the 3D reconstruction of TLL-2 monomer generated by angular reconstitution of the class sums are shown. (**D**) The dimeric TLL-2 can be compared and shows a similar conformation to the monomer. Images of the 3D reconstruction represented as a solid surface are shown.

**Figure 3:**
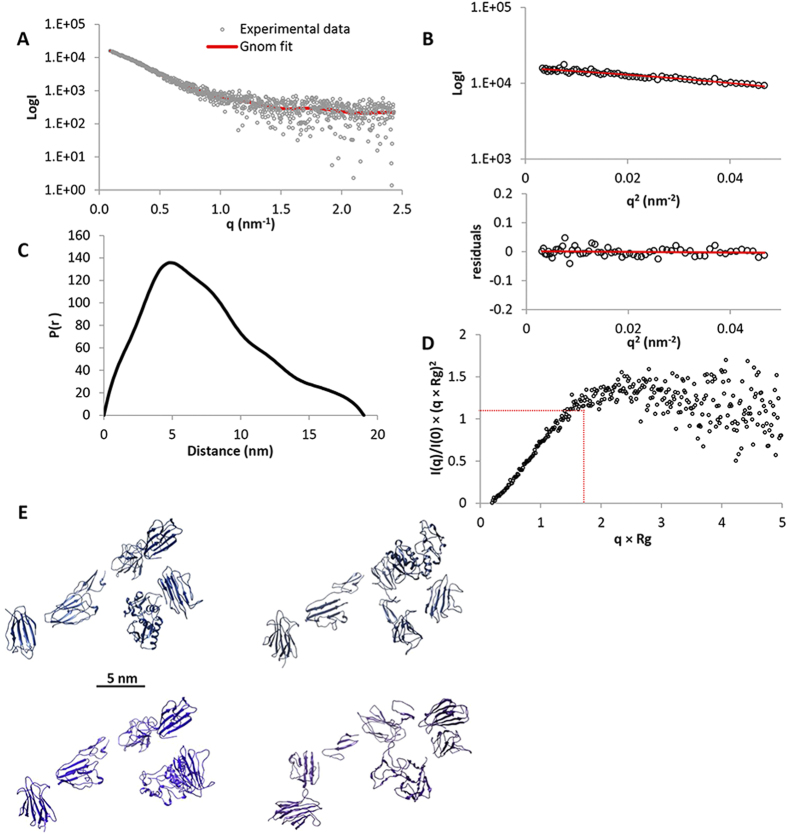
Solution small angle X-ray scattering data for TLL-2 in the absence of Ca^2+^. (**A**) The experimental SAXS data for TLL-2 in the presence of EDTA are plotted as a function of q. (**B**) The low-angle region of the X-ray scattering data is show in the form of a Guinier plot, which is linear. (**C**) The distance distribution function shows a maximum dimension of 19 nm. (**D**) The dimensionless Kratky plot is shown with peak at ~2.3 qxRg, the expected peak maxima for a globular protein is indicated in red. (**E**) Four representative rigid body models generated with SASREF are shown.

The structure of TLL-2 was investigated using single particle EM with negative staining (Fig. 2). There were two oligomeric states of TLL-2 observed on the EM grid, a monomeric form and a dimer, which was consistent with the shape seen for mTLD and TLL-1 previously^16^,^17^. Using reference-free methods, 3D reconstructions were calculated using angular reconstitution. The 3D model generated for the monomer was an elongated asymmetric shape with dimensions 17.2 nm × 6.9 nm × 6.9 nm (Fig. 2B). The dimer had a similar overall shape to the models produced for mTLD and TLL-1 and it was possible to segment the dimer into two units with consistent shape to the monomer (Fig. 2D). To determine the 3D shape of the protein in solution, SAXS measurements were made at the PETRAIII synchrotron radiation source (Fig. 3). In the presence of calcium, TLL-2 had a tendency to aggregate at high concentrations therefore SAXS data were collected in the presence of EDTA (Fig. 3A). The data quality was assessed using Guinier plots, to check for aggregation in the sample (Fig. 3B). The radius of gyration (Rg) obtained from the Guinier plot was 5.7 nm. The maximum particle dimension was estimated as 19 nm using indirect Fourier transform with GNOM (Fig. 3C)^19^. The dimensionless Kratky plot (Fig. 3D) shows that the protein is folded but not globular as there is a peak with maxima ~2.3, 1.4 rather than 1.73, 1.1 as expected for a globular protein^20^. Rigid body modeling was also performed using homology models of the eight TLL-2 domains in SASREF^21^. The models fitted the experimental data with a mean discrepancy factor *x* of 1.1 (N = 8; q < 2.5 nm^−1^). Each simulation produced fairly similar models with an asymmetric elongated shape (Fig. 3E).

**Figure 4:**
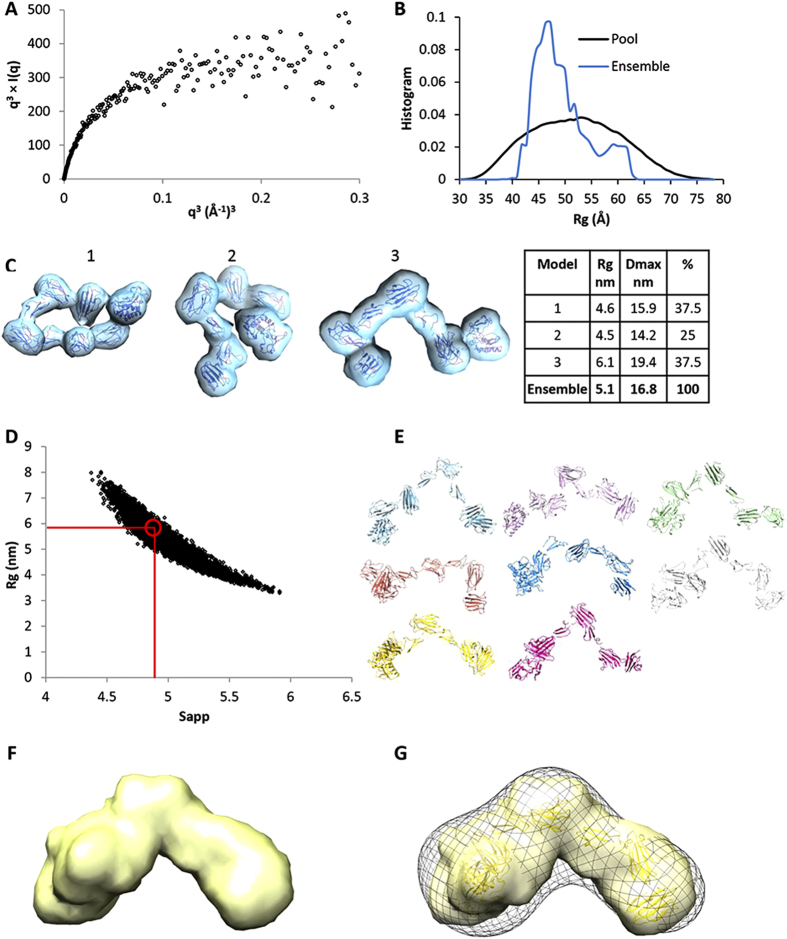
Flexibility in the absence of Ca^2+^ and conformation with Ca^2+^-bound. (**A**) SIBYLS plot shows a flexible protein in the absence of Ca^2+^. (**B**) Plot showing the distribution of the pool of models generated by EOM plotted as a function of Rg. The distribution of the ensemble selected from this pool is shown. (**C**) An ensemble of three structures generated EOM that represent the SAXS data. (**D**) Plot of the Rg vs S parameters of the Ranch models as calculated with SoMo. (**E**) Eight models that satisfy the hydrodynamic properties. (**F**) The average of these 8 models rendered to 20 Å resolution. (**G**) The average in (**F**) with one model fitted, superimposed with the EM map.

As these SAXS data were collected in the absence of Ca^2+^ which is known to stabilize cbEGF-CUB domain interfaces^22^, analysis of the flexibility of the protein was performed. For a non-flexible protein, the Porod-Debye plot (q^4^) should contain a plateau within the low resolution region of the SAXS data^23^, however no plateau was observed in the Porod-Debye plot (not shown). Furthermore, a plateau was present in the SIBYLS plot (q^3^) (Fig. 4A)^23^ which indicates a flexible protein. The Porod exponent, used to assess the compactness of the protein, was 2.4 which suggests that the protein is fairly elongated and is consistent with the AUC sedimentation parameters. The cross-sectional radius is 2.5 nm, significantly smaller than the Rg and Rh, which again is consistent with an elongated protein. To gain further insight into the apparent flexibility of TLL-2 in the absence of Ca^2+^ the conformational flexibility was evaluated using the ensemble optimization method (EOM)^24^. Since the structures of homologous domains are known, we were able to generate a pool of models that could be compared to the experimental scattering data (Fig. 4B). From a pool of 10,000 models an ensemble of three structures was predicted based on the SAXS data, two compact collapsed conformations with Rg of ~4.6 nm (62.5% of the ensemble) and a more elongated extended conformation with Rg 6.1 nm (37.5% of the ensemble) (Fig. 4C). The average R_g_ of 5.1 nm and D_max_ of 16.8 nm of the ensemble are similar to the estimated values by GNOM. The more elongated conformation bears most similarity to the EM structure, suggesting this most resembles the conformation in the presence of Ca^2+^.

### Hydrodynamic analysis of TLL-2 also supports an extended conformation in the presence of Ca^2+^

To further compare the predicted conformations with the experimental hydrodynamic data measured by MALS and AUC, a different approach was undertaken. First, the program Ranch^24^ was used to generate 5,000 random models using the eight individual domains of TLL-2. Following this, the program SoMo^25^ was used to calculate the hydrodynamic parameters (*s*, *R*_*h*_, *f/f*_0_ and *R*_*g*_) of each model (Fig. 4D). A subset of eight models that fitted the hydrodynamic data (*R*_*h,*_*s*) were identified (Fig. 4E). This procedure showed molecular shapes that are similar and an average model was generated rendered to 20 Å resolution (Fig. 4F) which fitted well with the EM data (Fig. 4G). These models also suggest that a significant degree of interdomain flexibility is possible in TLL-2, even in the presence of bound calcium (Fig. 4E).

### Deletion of CUB4 and CUB5 domains ablates TLL-2 chordinase activity

**Figure 5:**
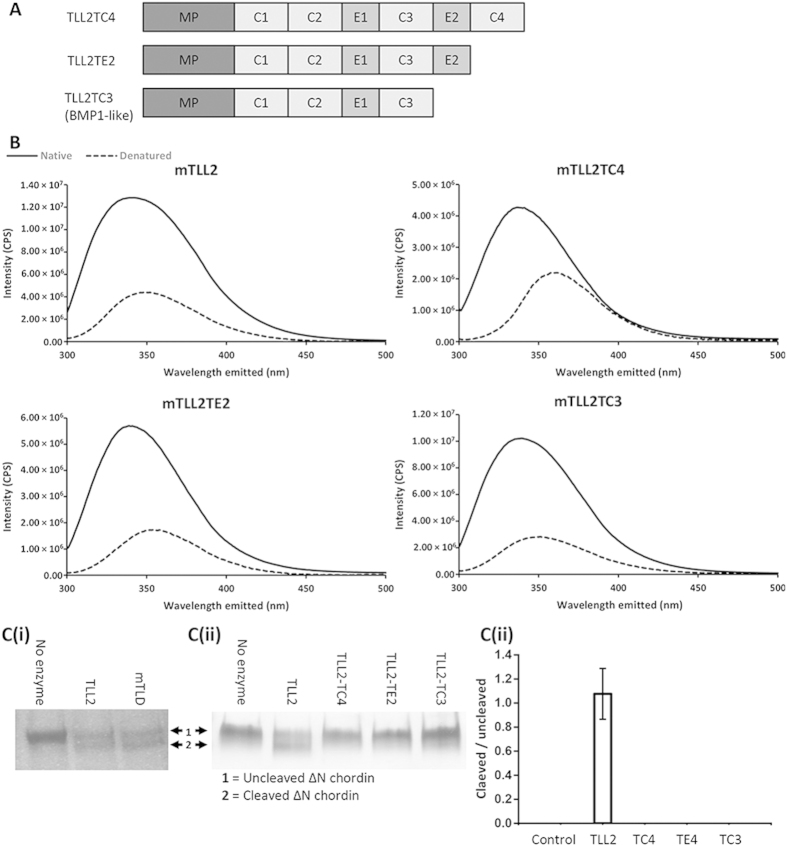
Unlike mTLD and TLL-1, CUB5 is necessary for TLL-2 chordinase activity. (**A**) Domain organisation of truncated forms of TLL-2 terminating after CUB4 (TLL2-TC4), EGF2 (TLL2-TE2) and CUB3 (TLL2-TC3), respectively. TLL2-TC3 is an analogue of BMP1. (**B**) Intrinsic fluorescence spectroscopy on TLL-2, TLL2-TC4, TLL2-TE2, TLL2-TC3 in native and denatured (8M Urea) conditions shows a shift in emittance indicating all proteins were folded. (Ci) SDS-PAGE analysis showing the cleavage of ΔN-chordin by TLL-2 and mTLD and (ii) TLL-2 compared to the truncated forms (TLL2-TC4, TLL2-TE2 and TLL2-TC3). This cleavage assay was carried out in triplicate and a representative gel is shown. (iii) Quantification of the cleavage assay by densitometry using the ratio of cleaved to uncleaved chordin. Errors from standard deviation (N = 3).

To investigate the role of the non-catalytic domains and determine whether TLL-2 is restricted by a substrate exclusion mechanism, TLL-2 variants lacking one or both C-terminal CUB domains (TLL2TC4 and TLL2TE2), and a construct with the same domain structure as BMP-1 (TLL2TC3) were made (Fig. 5A). The variants were analysed by SDS-PAGE and size exclusion chromatography to confirm in each case that the constructs were monomeric and intrinsic fluorescence spectroscopy to confirm that the proteins were folded (Fig. 5B). To determine the proteolytic activity of TLL-2 and variants, we compared their chordinase activity to that of mTLD (Fig. 5C). Chordinase activity was detected for TLL-2, this was similar to mTLD. However, the truncated forms of TLL-2 had no activity against chordin (Fig. 5C), unlike truncated TLL-1 and BMP-1 which are more active than their longer forms^16^,^17^ (i.e. full-length TLL-1 and mTLD). These assays suggest that CUB5 in TLL-2 is essential for chordin cleavage but is dispensable in TLL-1 and mTLD (i.e. in BMP1). This finding is consistent with studies on *Drosophila* TLD which also requires CUB5 for Sog cleavage^18^.

### Binding of non-catalytic domains from mammalian tolloid family to chordin.

**Figure 6:**
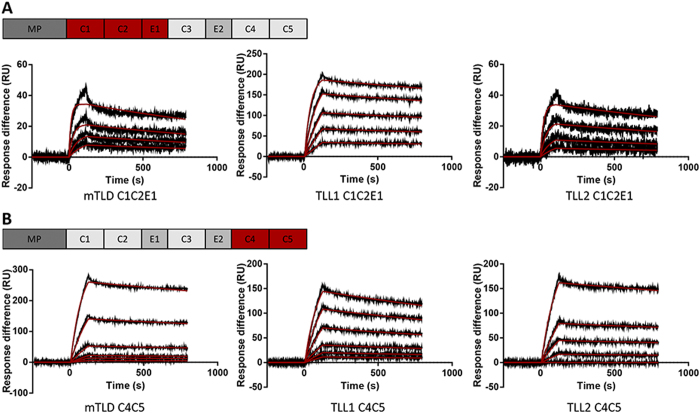
N- and C-terminal non-catalytic tolloid domains bind substrate tightly. SPR analysis of ΔN-chordin binding to CUB and EGF domains from mammalian tolloids. Real-time binding curves in black. Models of analysis in red. (**A**) Langmuir kinetic analysis of ΔN-chordin binding to immobilised protein fragments comprising CUB1, CUB2 and EGF1 (C1C2E1) from mTLD, TLL-1 or TLL-2. (**B**) Langmuir kinetic analysis of ΔN-chordin binding to immobilised protein fragments comprising CUB4 and CUB5 (C4C5) from mTLD, TLL-1 or TLL-2. Experiments performed in triplicate, representative curves shown.

Following from this finding, to determine if different non-catalytic domains are responsible for the interaction with chordin or if the affinity of the interaction differs between tolloids, we used SPR binding assays. The N-terminal non-catalytic domains (C1C2E1) from TLL-2, TLL-1 and mTLD/BMP1 were analysed for binding to ΔN-chordin (Fig. 6A). The K_D_ of each interaction was calculated by fitting a 1:1 Langmuir binding model to the data. All constructs bound with high affinity (e.g. TLL-1: K_D_ = 0.057 nM) (Table 1). The same assay was performed with the C-terminal non-catalytic domains, C4C5 from TLL-2, TLL-1 and mTLD (Fig. 6B). Again the binding of all three proteinases to chordin was high affinity, with K_D_s from 0.25 nM (TLL-2) to 0.056 nM (TLL-1). Together these data suggest a slightly stronger interaction between chordin and TLL-1 and weaker to mTLD/BMP1 and TLL-2 but in all cases binding was tight and in the sub-nanomolar range. Although CUB5 is required for TLL-2 cleavage of chordin, but dispensable in mTLD/TLL-1, the N-terminal non-catalytic domains also bind to chordin with high affinity.

## Discussion

Our previous studies had demonstrated that a substrate exclusion mechanism controls the activity of mTLD and TLL-1^16^,^17^. Calcium-dependent dimerisation of mTLD and TLL-1 was postulated to lower tolloid activity by either reducing the number of monomers available to bind substrate or reducing the substrate affinity of each protease molecule within the dimer (due to steric hindrance caused by the C-terminal non-catalytic domains partially occluding the adjacent protease domains). However, here we show that the third member of the long mammalian tolloid proteinase family, TLL-2 does not follow the same pattern as shown for mTLD and TLL-1. We report here that although some dimerisation does occur, mTLL-2 is predominantly a monomer in the presence of calcium (Figs 1 and 2). Consequently, the activity of mTLL-2 cannot be controlled by the same substrate exclusion mechanism as for mTLD and TLL-1.

We have previously shown that removing CUB and EGF domains from the C-terminus of either mTLD or TLL-1 increases the chordinase activity of these proteinases^16^,^17^. This finding is consistent with other reports that the protease domain alone of BMP-1 cleaved procollagen VII with greater efficiency than full-length BMP-1^26^ and that the protease domains of both TLL-1 and BMP1 cleaved probiglycan with greater efficiency than the corresponding full-length tolloids^14^. The present study demonstrates that removing the corresponding domains from the C-terminus of TLL-2 does not alter the fold of the truncated proteinase, yet ablates chordinase activity (Fig. 5), indicating that the deleted non-catalytic domains form critical interactions with the substrate. Consequently, our data suggest that substrate recognition is an important mechanism of TLL-2 activity. Similarly, *Drosophila* tolloid (dTLD) has also been found to have a requirement for CUB5 in order to cleave Sog, the chordin analogue in flies^18^.

The non-catalytic domains are certainly important in the activity and substrate specificity of tolloids. For example, Kadler and co-workers demonstrated that CUB2 was necessary for maximum cleavage of procollagen I by BMP-1^27^. Stocker and co-workers analysed the binding of CUB and EGF domain doublets to procollagen I by SPR and showed that binding affinity to procollagen increases for domains towards the C-terminal end of mTLD^28^. They also show that fragments containing EGF domains bind procollagen more strongly than those containing only CUB domains. However, we show that the CUB4-CUB5 region binds very tightly to chordin so EGF domains appear not to be a requirement for tolloid-chordin binding. A previous study using BMP1 and TLL-2 domain swaps showed that the protease domain of TLL-2 fused to the non-catalytic domains of BMP1 was able to cleave chordin as efficiently as BMP1 demonstrating that the non-catalytic domains are essential for activity. Indeed the protease domain of BMP1 fused to the non-catalytic domains of TLL-2 had no chordinase activity and in addition, the protease domain of TLL2 was unable to cleave procollagen even when fused to the CUB domains of BMP1 showing that the non-catalytic domains also strongly influence substrate specificity^15^.

In this study, we have shown that protein constructs from all mammalian tolloids, comprising either CUB1-CUB2-EGF1 or CUB4-CUB5, bind to chordin (Fig. 6). This observation indicates that when a tolloid proteinase interacts with chordin, it makes protein-protein interactions that span the majority of the non-catalytic domains. These interactions may be necessary in order to bring both proteinase and substrate into a favourable conformation for cleavage to occur. With respect to TLL-2, since CUB5 is required for chordin cleavage, a specific interaction between CUB5 and chordin may be necessary to enable cleavage by the proteinase. The extended, flexible conformation of TLL-2, indicated by the biophysical data (Figs 3 and 4), suggests that TLL-2 has the potential to change conformation upon substrate binding which may be mediated by the non-catalytic CUB and EGF domains.

TLL-2 must interact with the substrate chordin in a different way to the other members of the tolloid family. This difference is conferred by two factors. Firstly, TLL-2 is predominantly a monomer in the presence of calcium, whereas mTLD and TLL-1 are calcium dependent dimers. The TLL-1 dimer has a strong intermolecular interaction (<nM Kd) which is a similar affinity to the tolloid-chordin interaction we observe, whereas the mTLD intermolecular dimer interaction is weaker (μM Kd). Secondly, the non-catalytic domains of TLL-2 show differences to the corresponding non-catalytic domains of mTLD and TLL-1. CUB5 of TLL-2 is necessary for chordinase activity, whereas removal of C-terminal CUB domains from mTLD and TLL-1 increases chordinase activity. Similarly for dTLD, in the absence of CUB4 or CUB5 dTLD was unable to bind Sog (as assessed by immunoprecipitation)^18^. However, dTLD also has further differences to the mammalian tolloids, for example the requirement of dpp (BMP) ligand for Sog cleavage^29^. Collagen IV also provides a scaffold for the Sog-tolloid interaction^18^ whereas the mammalian tolloid family do not appear to require these other contributors. Differences between mTLD/TLL-1 and TLL-2/dTLD may point to a discrete range of substrates processed by TLL-2/dTLD, where the requirement could be for specialised substrate cleavage rather than the broad specificity seen for TLL-1/BMP1. Analysis of TLL-2, the third member of the mammalian tolloid proteinases, has provided an insight into how substrate recognition and substrate cleavage by tolloids could be mediated by the non-catalytic domains of these proteinases.

## Materials and Methods

### Expression and purification of recombinant proteins

Full length human TLL-2^15^ was used to generate constructs encoding TLL2-TC4, TLL2-TE2 and TLL2-TC3 by PCR. The constructs were ligated into a modified pCEP-Pu vector^30^ and transfected into HEK 293-EBNA cells cultured as described previously^16^. Constructs encoding mTLD-C1C2E1, TLL1-C1C2E1, TLL2-C1C2E1, mTLD-C4C5, TLL1-C4C5 and TLL2-C4C5 were generated at the Oxford Protein Production Facility (OPPF-UK), Harwell, UK in a pOPINTTGneo expression vector^31^ provided by OPPF and transfected into HEK 293S cells. ΔN-chordin was generated as previously described^32^. For all constructs a 6x histidine tag was incorporated at the C-terminus. Conditioned media was concentrated and buffer exchanged into 10 mM HEPES, 500 mM NaCl, 10 mM imidazole, 2 mM CaCl_2_, pH 7.4 using tangential flow ultrafiltration (Pall Life Sciences). All recombinant proteins were then purified by nickel affinity chromatography followed by size-exclusion chromatography on an AKTA purifier HPLC using a Superdex200 10/300GL column (GE Healthcare) in 10 mM HEPES, 500 mM NaCl, 2 mM CaCl_2_, pH 7.4. Where needed, proteins were concentrated using Vivaspin centrifugal concentrators (Sartorius). Protein identities were confirmed by in-gel trypsin digestion and liquid chromatography tandem mass spectrometry (LC-MS/MS) using a NanoAcquity LC (Waters) coupled to a LTQ Velos (Thermo Fisher Scientific).

### Intrinsic fluorescence spectroscopy (IFS)

Samples (0.25 μM) in a 16.100-F quartz cuvette (Starna Scientific, Essex, UK) were analysed using a JASCO FP-750 Spectrometer (JASCO UK, Essex, UK) under the control of the Spectra Manager software. The excitation wavelength was 290 nm and an emission scan between 300–500 nm was recorded. Analyses were repeated following addition of 8 M urea (final concentration) to each sample. Spectrums generated by buffers alone were subtracted from the protein measurements.

### Multi-angle light scattering (MALS) Analysis

Samples (0.5 ml at approximately 0.5 mg/ml) were loaded onto a Superdex200 10/300GL column running at a flow rate of 0.75 ml/min in 10 mM Tris, 500 mM NaCl, 2 mM CaCl_2_, pH 7.4. Samples eluting from the column passed through a DAWN Wyatt HeliosII 18-angle laser photometer. One of the detectors on the photometer was replaced with a Wyatt QELS detector. This was coupled to a Wyatt Optilab rEX refractive index detector and the molecular mass moments, polydispersity, hydrodynamic radii and concentrations of the resulting peaks were analysed using Astra 6.1 (Wyatt, Santa Barbara, USA).

### Analytical Ultracentrifugation (AUC)

Sedimentation velocity AUC on TLL-2 (0.4 mg/ml) was carried out at 45,000 rpm, 20 °C using an XL-A ultracentrifuge with an An50Ti-4-hole rotor (Beckman Coulter, High Wycombe, UK). Sedimentation was scanned every 90 seconds for 200 scans. Data were analysed using Sedfit^33^.

### Solution Small angle X-ray scattering (SAXS)

SAXS intensity data on TLL-2 in 10 mM Tris, 500 mM NaCl, 2 mM EDTA pH 7.4 at 2 mg/ml were collected from protein samples and matched buffer blanks at the EMBL-P12 beamline at PETRAIII (DESY, Hamburg) employing automated data acquisition and radial averaging protocols^34^. The forward scattering intensity, Rg and distance distribution function p(r) were evaluated with GNOM^19^. Rigid body modelling against the experimental SAXS data was performed with SASREF^21^ using the eight individual domains of TLL-2 with a distance range of 5–10 Å between each domain. EOM^24^ was used to evaluate the flexibility of TLL-2 and to select the best fitting ensemble to the SAXS data.

### Hydrodynamic analysis

A library of 5,000 conformers of TLL-2 were generated by Ranch^24^ then SoMo^25^ was used for calculation of hydrodynamic parameters for each model including (*R*_*g*_), hydrodynamic radius (*R*_*h*_), sedimentation coefficient (*s*), maximal linear distance (*D*_max_) and frictional ratios. Experimental hydrodynamic data from MALS and AUC were used to select a pool of models that fit the experimental values.

### EM and Single-Particle Analysis

TLL-2 (~10 μg/ml) in 10 mM HEPES, 500 mM NaCl, 2 mM CaCl2, pH 7.4 was adsorbed onto glow-discharged carbon-coated grids and stained with 4% (w/v) uranyl acetate (pH 4.7). Grids were observed using either a FEI Tecnai Twin (120 keV) or Biotwin (100 keV) transmission EM for the monomer and dimer, respectively. Images were recorded under low dose conditions (<10 e^−^/Å^2^) on 2,048 × 2,048 pixel CCD cameras at 30,000 × (2.8 Å/pixel monomer) and 28,000 × magnification (3.5 Å/pixel dimer) between −0.2 and −2.0 μm defocus. Eman2^35^ was used for particle picking and image processing. Images were CTF corrected and the total number of particles in each the dataset was 1,260 for TLL-2 monomers and 5,524 for TLL-2 dimers. Characteristic class-sum images were used as references to align the dataset. Angular reconstruction produced unique projection classes, enabling calculation of an initial 3D reconstruction which was then subjected to 5 rounds of iterative refinement. C2 symmetry was applied to the TLL-2 dimer and a 20 Å low-pass Gaussian filter was used at each stage of the refinement.

### Activity Assays

Purified ΔN-chordin (1.8 μg) was incubated in the presence or absence of 110 ng enzyme in 50 mM Tris-HCl (pH 7.4) containing 150 mM NaCl and 5 mM CaCl_2_ at 37 °C for 16 hours^36^. Reactions were stopped by adding LDS sample buffer (Life Technologies) and 2.5 % β−mercaptoethanol and heating to 95 °C for 5 min. Reaction products were separated by SDS-PAGE and visualised by silver staining. Chordinase assay products were quantified by densiometry using GeneTools software (Syngene UK, Cambridge, UK).

### Surface plasmon resonance (SPR)

Protein-protein interactions were measured with a ProteOn XPR36 (Bio-Rad Laboratories) in 10 mM HEPES, 150 mM NaCl, 2 mM CaCl_2_, 0.05% Tween 20, pH 7.4 (running buffer) at 25 °C. Proteins were immobilised on a GLC chip (Bio-Rad Laboratories) via amine coupling and a reference lane blocked with ethanolamine. Analytes diluted in running buffer were injected onto the surface of the chip at increasing concentrations for kinetic analysis. Response curves were analysed using the ProteOn Manager Software (Bio-Rad Laboratories) fitting a 1:1 Langmuir model to each interaction.

## Additional Information

**How to cite this article**: Bayley, C. P. *et al.* Diversity between mammalian tolloid proteinases: Oligomerisation and non-catalytic domains influence activity and specificity. *Sci. Rep.* **6**, 21456; doi: 10.1038/srep21456 (2016).

## Figures and Tables

**Table 1:** Kinetic analysis of ΔN-chordin binding to different fragments of mammalian tolloids, obtained by SPR.

Immobilised tolloid fragment	K_D_ (nM) Langmuirbinding
mTLD CUB1-CUB2-EGF1	0.278 ± 0.85
TLL1 CUB1-CUB2-EGF1	0.057 ± 0.003
TLL2 CUB1-CUB2-EGF1	0.247 ± 0.26
mTLD CUB4-CUB5	0.19 ± 0.02
TLL1 CUB4-CUB5	0.056 ± 0.01
TLL2 CUB4-CUB5	0.25 ± 0.02

Experiments performed in triplicate.
